# Contusion of Popliteal Artery without Damage of the External Parts, Followed by Thrombosis and Death

**Published:** 1882-08

**Authors:** 


### CONTUSION OF THE POPLITEAL ARTERY WITHOUT DAMAGE OF THE EXTERNAL PARTS, FOLLOWED BY THROMBOSIS AND DEATH.

A man, thirty-two years of age, was run over by an omnibus and brought to the Hospital Necker. The wheels of the omnibus ran over the lower limbs just below the knee joints. The right limb was seriously damaged, and there was a good deal of bleeding, which was stopped by a tampon. On the left limb there was a large ecchymosis, without any damage of the external parts. Both knee joints were infiltrated and immovable. On the following night there was noticed considerable friction sound under the knee joint. Both limbs were treated antiseptically. The pulse was 100; temperature 86, and the patient vomited. The next day all the symptoms were aggravated; temperature 80. The left limb was cold, insensible, and no arterial pulsations. The patient vomited severely. The next day gangrene of the left limb set in. Antiseptic treatment was abandoned on account of the dark-colored urine. An operation was not determined upon, on account of the severe general symptoms (sic!). The patient died after being five days in the hospital. The autopsy showed a bi-malleolar fracture of the right limb and bloody infiltration. On the left limb the lesions were highly interesting. The articulation of the knee joint was filled with blood. The subcutaneous tissue was largely infiltrated with blood, and the infiltration was dispersed through the muscular interstices down to the foot. The soleus muscle was partly ruptured, and torn from its superior insertion. The popliteal artery was not ruptured. On the posterior aspect of the tibia the popliteal artery appeared to be blackened, and cylindrical in form. The external wall of the artery was infiltrated with blood, and there was a complete occlusion, the artery forming a flat cylinder. This was caused by a contusion of the artery, which had been pressed between the tibia and the wheels of the omnibus. On opening the artery there was a dark clot three cm. long, adhering to the anterior walls of the vessel. The inner coats were ruptured, and the adventitia infiltrated with blood. The aorta, the femoral artery and the sigmoid did not show any signs of atheroma.—*France Médicale.*

---

"I don't, myself, like living in a world with such a multitude of murdered men in the ground of it—though we *are* making heliotropes of them, and scientific flowers that study the sun. I wish the scientific men would let me and other people study it with our own eyes, and neither through telescopes nor heliotropes." (Ruskin.)

